# A label-free LC/MS-based enzymatic activity assay for the detection of PDE5A inhibitors

**DOI:** 10.3389/fchem.2023.1097027

**Published:** 2023-02-13

**Authors:** Yufeng Ma, Fengsen Zhang, Yijing Zhong, Yongchun Huang, Yixizhuoma, Qiangqiang Jia, Shoude Zhang

**Affiliations:** ^1^ State Key Laboratory of Plateau Ecology and Agriculture, Qinghai University, Xining, China; ^2^ Department of Pharmacy, Medical College of Qinghai University, Xining, China

**Keywords:** PDE5A, LC/MS, cGMP, enzyme activity, inhibitor

## Abstract

Phosphodiesterase type 5 (PDE5), a cyclic nucleotide phosphodiesterase, controls the duration of the cyclic guanosine monophosphate (cGMP) signal by hydrolyzing cGMP to GMP. Inhibiting the activity of PDE5A has proven to be an effective strategy for treating pulmonary arterial hypertension and erectile dysfunction. Current enzymatic activity assay methods for PDE5A mainly use fluorescent or isotope-labeled substrates, which are expensive and inconvenient. Here, we developed an LC/MS-based enzymatic activity assay for PDE5A without labeling, which detects the enzymatic activity of PDE5A by quantifying the substrate cGMP and product GMP at a concentration of 100 nM. The accuracy of this method was verified by a fluorescently labeled substrate. Moreover, a new inhibitor of PDE5A was identified by this method and virtual screening. It inhibited PDE5A with an IC_50_ value of 870 nM. Overall, the proposed strategy provides a new method for screening PDE5A inhibitors.

## 1 Introduction

**FIGURE 1:**
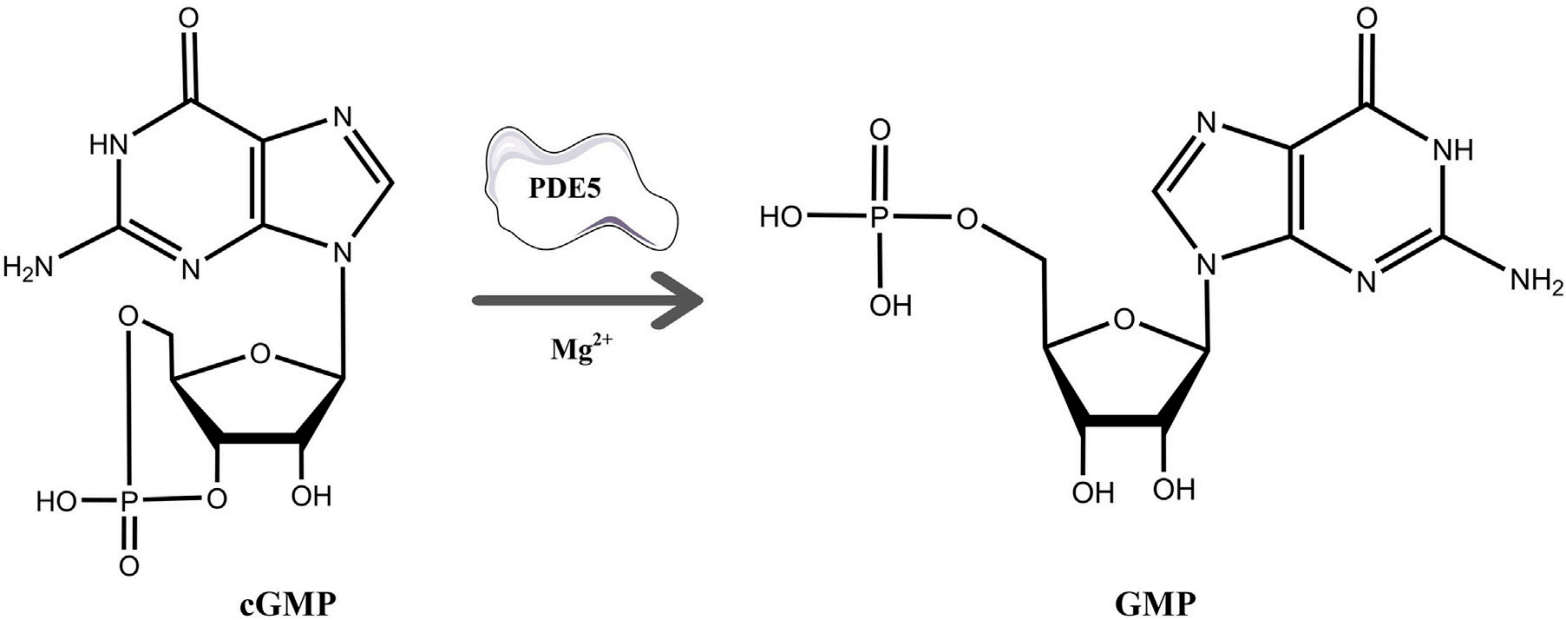
The process of hydrolysis of cGMP to GMP by PDE5A and differences in the chemical structures of cGMP and GMP.

cGMP is a unique second messenger that is commonly involved in the opening of cell membrane ion channels (Biel and Michalakis 2009), glycogen decomposition (Zhang et al., 2022), apoptosis (Kim et al., 1999), and relaxation of smooth muscle (Ignarro and Kadowitz 1985). It is produced by soluble guanosine cyclase (sGC) and granular guanosine cyclase (pGC) after combining with nitric oxide (NO) and natriuretic peptides (NPs), respectively (Cerra and Pellegrino 2007; Hofmann 2020; Friebe et al., 2020). cGMP mediates NO biological signals through three major cellular targets, including cGMP-dependent protein kinase G (PKG), cyclized nucleotide cation-gated channels (CNG), and PDEs (Francis et al., 2010). The main target molecule of cGMP action is PKG, and the activation of PKG is usually associated with the regulation of processes such as calcium homeostasis (Chen et al., 2009), smooth muscle contraction (Rybalkin et al., 2003), platelet activation, and adhesion (Li et al., 2003). The intracellular homeostasis of cGMP is mainly regulated by PDE5A and GC (Mullershausen et al., 2004). The PDE superfamily can be divided into 11 families, namely, PDE1-11, according to their sequence homology, substrate specificity, and regulatory characteristics (Omori and Kotera 2007). PDE5A is the most well-studied phosphodiesterase in this family and is expressed in lung, brain, kidney, cardiomyocytes, gastrointestinal tissues, vascular smooth muscle cells, platelets, penile spongy body, and many other tissues (Kotera et al., 2000; Daniela et al., 2001). As shown in Figure 1, PDE5A converts cGMP to 5′-GMP, an inactive form, by hydrolyzing the phosphodiester bond (Lin 2004; Lugnier 2006). Preventing the degradation of cGMP by PDE5A inhibitors, such as sildenafil and vardenafil, has become a strategy for the treatment of diseases such as pulmonary hypertension and erectile dysfunction (Corbin et al., 2005; Sandner et al., 2007). However, due to the high homology among family members, the clinical application of drugs is limited (Setter et al., 2005; Ueda et al., 2019), so the development of selective PDE5A inhibitors is of great importance.

Fluorescent or isotope-labeled substrates are widely used in current inhibitor screening for PDE5A (Xu et al., 2011; Li et al., 2013; Shibata et al., 2020) and are expensive or inconvenient. Here, we developed a novel PDE5A enzymatic activity assay method based on LC/MS. In this method, free-labeled cGMP and GMP are separated by UHPLC with high resolution and quantified by mass spectrometry at the nanomolar level. The enzymatic activity of PDE5A treated with inhibitor or without inhibitor could be detected by analyzing the quantity change in cGMP or GMP. In this work, we not only confirmed the accuracy of this method with a fluorescent labeling method but also verified a virtual screening result of PDE5A inhibitors with this method. Finally, a new PDE5A inhibitor was identified with this method.

## 2 Materials and methods

### 2.1 Materials

The following materials were obtained from Sigma–Aldrich (St. Louis, MO, United States): guanosine 3′,5′-cyclic monophosphate sodium salt (cGMP, HPLC, 99%), zirconyl chloride octahydrate (ZrOCl_2_, reagent grade, 98%), and bovine serum albumin (BSA, ≥98%). Guanosine monophosphate (GMP), sildenafil citrate (HPLC), and vardenafil HCl trihydrate (HPLC) were purchased from Aladdin (Shanghai, China). Deionized water was purified by a Milli-Q purification system (Millipore, Bedford, MA, United States). HPLC grade acetonitrile, HPLC formic acid, HPLC methanol, and HisPur™ Ni-NTA Resin were purchased from Thermo Fisher Scientific (Vilnius, Lithuania). Dimethyl sulfoxide (DMSO≥99%), ultrafiltration spin columns (0.5 mL, 10 kDa MWCO, PES, Sartorius), and 96-well black opaque plates were purchased from Beyotime Biotechnology (Shanghai, China). Proanthocyanidins and nine other standards were purchased from Baoji Herbest Bio-Tech Co., Ltd, and 2′-O-(6-[tetramethylrhodaminyl]aminopentylcarbamoylethylcarbonyl)guanosine-3′,5′-cyclic monophosphate trifluoroacetate salt (96%) was purchased from AAT Bioquest, Inc. (TAMRA-R-cGMP, Sunnyvale, CA, United States). All other reagents were of analytical grade and obtained from Sinopharm Chemical Reagent Co., Ltd. (Shanghai China).

### 2.2 Protein expression and purification

The catalytic domain of PDE5A (residues 535-862; GenBank accession number BC126233.1) was subcloned into the T7 promoter-driven expression vector pET21b with a 6 × His-tag at the C-terminus (Hsieh et al., 2020). The recombinant plasmid was transformed into *E. coli* strain BL21 (DE3) and grown in an autoinducing medium (Studier 2005) containing 50 μg/mL ampicillin at 37 C until OD600 = 0.6–0.7, then induced protein expression at 15°C for 40 h. The PDE5A protein was purified through the Ni-NTA column (Thermo Scientific) and further purified by the HiPrep™26/60 Sephacryl™-S-200HR column (GE Healthcare). A typical batch cell yielded over 10 mg of the PDE5A protein from 1 L of autoinducing medium with a purity >95% based on SDS‒PAGE. The protein was concentrated to a certain concentration using centrifugal filters and stored in a storage buffer (50 mM NaCl, 20 mM Tris-HCl pH 7.5, 1 mM β-mercaptoethanol, 1 mM EDTA, and 5% glycerol).

### 2.3 LC/MS method

#### 2.3.1 LC/MS condition

The chromatographic separation of cGMP and GMP was achieved on a Hypersil GOLD™ aQ C18 column (1.9 μm, 100 mm × 2.1 mm, Thermo Scientific) using phase A (100% LC/MS grade acetonitrile) and phase B (0.1% LC/MS grade formic acid in water), followed by 0.00 min: 1% A, 3 min: 1% A, 6 min: 90% A, 8 min: 90% A, 10 min: 1% A, 12 min: 1% A, at a consistent flow rate of 0.3 mL/min. The injection volume was set at 2 μL, and the column temperature was controlled at 35°C.

MS analysis was performed on a Thermo Fisher Q Exactive Plus mass spectrometer (Waltham, MA, United States; Thermo, Bremen, Germany) with a heated electrospray ionization (HESI) ion source. The separated samples from UHPLC were injected into the system and analyzed by positive ion swing with full MS and SIM scans. The HESI parameters in positive polarity were as follows: spray voltage 3.5 kV; capillary temperature 320°C; auxiliary gas heater temperature 300 C; sheath gas flow rate 35 μL/min; auxiliary gas flow rate 10 μL/min; S-lens RF level was 50 V; full MS scan resolution of primary parent ions was 70,000 full width at half maximum (FWHM); scan range was 100–1,500 m/z; the automated gain control (AGC) target was 1 E^6^; SIM scan resolution of daughter ion was 35,000 FWHM; maximum IT was 50 m; isolation window in quadrupole was 3.0 m/z, and specific normalized collision energy (NCE) for each precursor m/z in 35.

During analyzation, Xcalibur 4.1 (Thermo Fisher Scientific, San Jose, CA, United States) was used to determine and integrate peak areas. After that, the linear correlation between the peak area and concentration of GMP was calculated based on the above condition and gradient concentration of GMP.

#### 2.3.2 Method validations

Method validation was performed from the aspects of linearity, range, precision accuracy, reproducibility, stability according to our previous research (Jia et al., 2020). Briefly, the linearity was expressed by linear regression between the peak area and the analyte concentration. LOD was defined as the concentration when the signal intensity was three times than that of noise (S/N = 3) and LOQ was defined as the concentration when S/N = 10. To determine the accuracy, reaction samples were spiked with standards solution of cGMP and GMP with a concentration of 5 μg/mL and analyzed six times for each standard and the recovery rate was calculated by comparing the changes of amounts. The precision was detected in a day (intra-day) and in 3 days (inter-day) with the cGMP and GMP standards at the concentration 5 μg/mL. The stability was evaluated by analyzing the 5 μg/mL mixed standard solution at 0, 12, 24, 36, and 48 h, and the variations were expressed as RSDs. The cGMP and GMP standards at the concentration of 5 μg/mL were used to determine the repeatability.

### 2.4 Enzymatic activity assay method

Enzyme activity refers to the ability of an enzyme to catalyze a certain chemical reaction. The enzyme activity of PDE5A can be calculated by the increase in the product GMP per unit time (U/mg, where 1 U is the amount of enzyme that consumes 1 μmol of cGMP per minute). Plots of GMP production vs. time and amount of enzyme vs. reaction speed were fit by linear regression using GraphPad Prism 9. The absolute value of the slope of the production vs. time curves is defined as the activity of PDE5A. Relationships between substrate concentration [*S*] and PDE5A activity (*v*) were fit by non-linear regression using GraphPad Prism 9 according to the Michaelis and Menten equation (Bisswanger 2014),

$$v=v_{\max}\left[S\right]/\left(Km+\left[S\right]\right)$$ (1)

where $v_{\max}$ is the maximum reaction rate and *K*_m_ the concentration of substrate [*S*] at which the enzymatic reaction reaches half the maximum velocity $v_{\max}$ . The total reaction volume was set to 200 μL, and the substrate and enzyme were each 100 μL. The reaction was terminated by boiling for 5 min at 100°C. After cooling, the protein was removed by ultrafiltration membrane (0.5 mL, 10 KD, Merck Millipore) filtration, and the filtrate was detected by LC/MS.

### 2.5 Assay for measuring PDE5A inhibition

Each assay was performed in a 150 μL reaction volume containing PDE5A (50 nM), various concentrations of compounds, and cGMP (10 μM), each 50 μl PDE5A was placed at room temperature for 30 min with the small molecule, cGMP was added, and the reaction mixture was left to settle at room temperature for 15 min. For the compound inhibition study on PDE5A, stock solutions of the compounds were prepared in 100% DMSO and diluted in reaction buffer (10 mM Tris–HCl pH 7.5, 0.1 mg/mL BSA, 10 mM MgCl_2_, and 1 mM β-ME) to the appropriate concentrations to give a final concentration of <2% DMSO. After the reaction, the mixed solution was filtered by an ultrafiltration membrane to remove the protein, the filtrated solution was tested by LC/MS using established methods, and the GMP peak release was recorded. Relative PDE5A activity (%) was calculated by normalizing the activity of negative controls (background factors need to be excluded). PDE5A inhibitory activity was calculated from the equation below (background subtraction for each group: $C_{negative.GMP}$ ).

$$\%PDE5A\;inhibition=\left(C_{positive.GMP}-C_{compound.GMP}\right)/C_{positive.GMP}\times 100\%$$ (2)

In this equation, $C_{negative.GMP}$ represents the production of GMP (without enzyme); $C_{positive.GMP}$ indicates the amount of product after hydrolysis of cGMP by PDE5A; and $C_{compound.GMP}$ represents the amount of hydrolysate after the compound inhibits PDE5A.

### 2.6 Virtual screening method

An in-house compound database containing 1,427 natural products was used for virtual screening. The structures and X-ray crystal structure of PDE5A (PDB: 1TBF) were prepared by MGLTools developed by the Scripps Research Institute. The grid box of the receptor was centered on the ligand sildenafil in the refined crystal structure and defined to enclose the residues located within 40 points from the ligand. The docking process was performed using AutoDock Vina with the default docking parameter (Trott and Olson 2010). Ten conformers were generated in the docking process for every compound, and only the top conformer for each compound was retained. Finally, the top 50 compounds were reserved for further visual observation, and 10 compounds were selected for experimental testing.

### 2.7 Enzyme activity assay based on fluorescently labeled substrate

TAMRA-R-cGMP is a derivative of cGMP linked with a red fluorescence group. It can bind to cGMP binding site of PDE5A and has been used for enzyme activity determination and inhibitor screening according to changes in fluorescence intensity (Santillo and Mapa 2018). Assays were performed in 96-well black opaque plates with a volume of 100 μL per well. Then, 200 nM PDE5A (25 μL) was mixed with various concentrations of compounds (25 μL) in reaction buffer (10 mM Tris–HCl pH 7.5, 0.1 mg/mL BSA, 10 mM MgCl_2_ and 1 mM β-ME). After incubation for 30 min at room temperature, an equal volume of 100 μM ZrOCl_2_ (25 μL) and 5 μM TAMRA-R-cGMP (25 μL) was added to the solution. Check for fluorescence quenching after 15 min. Incubations for negative control (no PDE5A), positive control (PDE5A), and background (assay buffer) were tested in triplicate wells for each assay run. The inhibition activity of the compounds was initially screened at 20 μM and the IC_50_ values for the active compounds were calculated at various concentrations. Fluorescence intensity was measured with Filter Max F5 Multi-Mode Microplate Readers (Molecular Devices) at an excitation wavelength of 535 nm and an emission wavelength of 590 nm. Each measurement was repeated at least 3 times, and IC_50_ values were calculated by non-linear regression in GraphPad Prism 9. PDE5A inhibitory activity was calculated from the equation below:

$$\%PDE5A\;inhibition=\left({FI}_{compound}-{FI}_{positive}\right)/\left({FI}_{negative}-{FI}_{positive}\right)\times 100\%$$ (3)

In this equation, ${FI}_{negative}$ represents the reaction system containing only fluorescent substrates (without enzyme); ${FI}_{positive}$ indicates the fluorescence value of the substrate after hydrolysis of cGMP by PDE5A; and ${FI}_{compound}$ represents the fluorescence value of the remaining substrate after the compound inhibits PDE5A.

## 3 Results and discussion

### 3.1 Establishment of the LC/MS-based enzymatic activity assay for the detection of PDE5A inhibitors

#### 3.1.1 Strategy of LC/MS-based enzymatic activity assay for the detection of PDE5A

**FIGURE 2:**
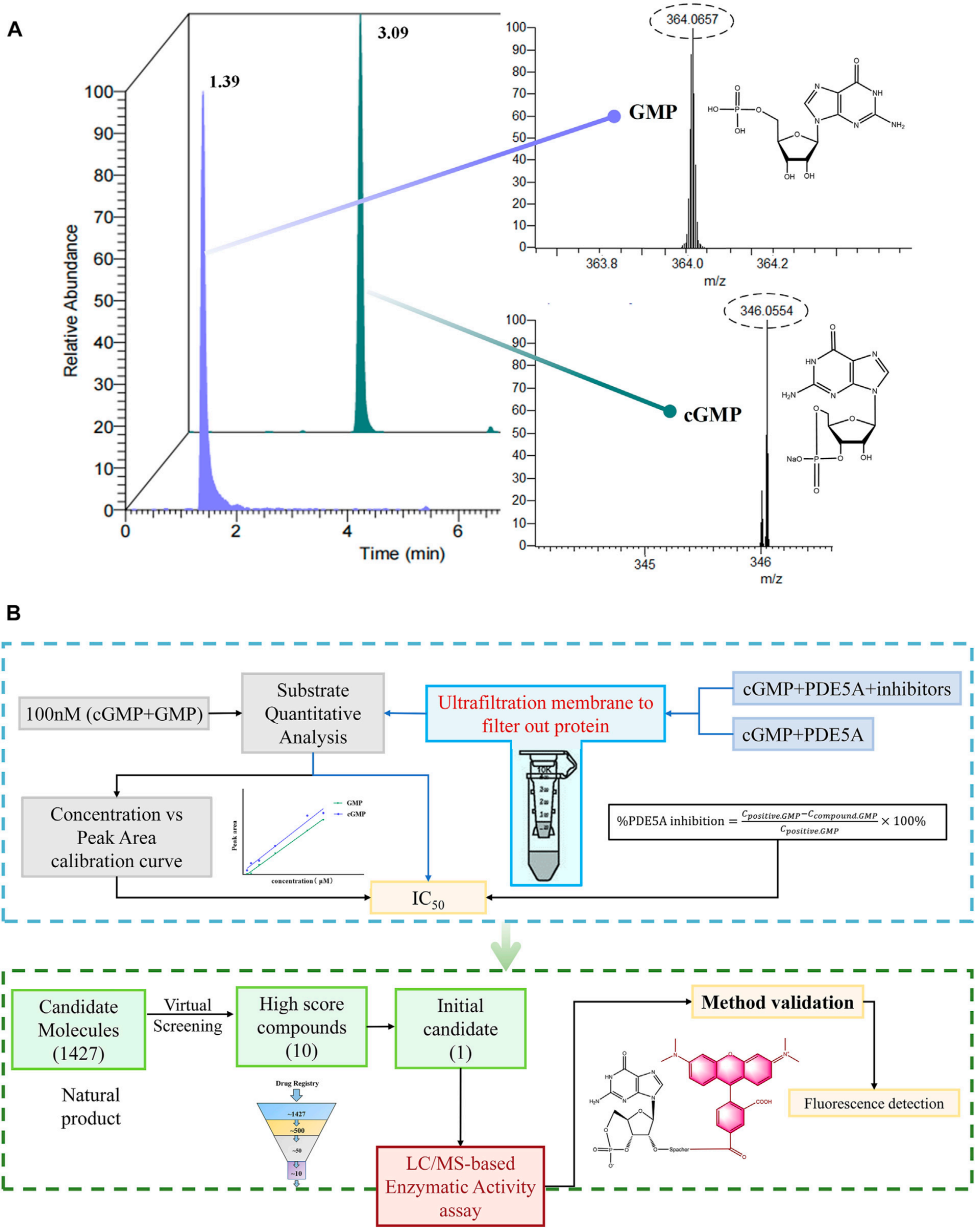
Enzyme activity assay and inhibitor screening strategy for PDE5A based on LC/MS. **(A)** The analyzed results of GMP and cGMP by LC/MS at 100 nM. **(B)** Routes for the determination of enzyme activity and new inhibitors of PDE5A by this method.

As shown in Figure 1, cGMP and GMP have different structures and molecular weights, which lead to different retention times in liquid chromatography. Based on our established liquid chromatography conditions, cGMP and GMP can be separated with high resolution, and the retention times were 3.09 min and 1.39, respectively (Figure 2A). After separation, cGMP and GMP can be further quantified by mass spectrometry. Based on this principle, we developed a new strategy to detect quantitative changes in cGMP and GMP in the catalytic reaction of PDE5A (Figure 2B). Both the decrease in cGMP and the increase in GMP are related to the activity of PDE5A. After the quantified PDE5A and cGMP were incubated with the compounds, PDE5A was removed from the reaction buffer by an ultrafiltration tube, and the filtrate was used to detect the changes in the cGMP and GMP contents. The ion peaks of cGMP and GMP could be extracted by Xcalibur 4.1 software and quantified by the standard curve between the GMP concentration and peak. The inhibition rate was calculated by Equation (B). Next, this method is used not only for the determination of the enzyme activity of PDE5A but also for the screening of new inhibitors in combination with virtual screening.

#### 3.1.2 Method validation

The results of method validation are summarized in Table 1. Within the concentration range, the calibration curves of the GMP and cGMP showed good linearity, with correlation coefficients greater than 0.9960. The LOD and LOQ ranges of the GMP and cGMP are 0.03–0.10 μg/mL and 0.02–0.05 μg/mL, respectively, which indicates that the detection of the GMP and cGMP by this method is sufficiently sensitive. The recovery range of accuracy for GMP and cGMP are 98.67% and 96.94, respectively, with RSD less than 3.79%. The RSDs of inter-day and intra-day precision ranged from 3.86% to 4.33% and from 2.01% to 3.89%, respectively. The RSD values in stability are less than 1.96%, which implied that GMP and cGMP in solution are stable for 48 h at room temperature. The RSD values of the repeatability ranged from 2.19% to 2.95%, which indicated that the developed method is reliable.

**TABLE 1:** Linearity, LOD and LOQ, accuracy, precision, stability and repeatability of the LC/MS method (*n* = 6).

Analytes	Linearity			LOD (μg/L)	LOQ (μg/L)	Accuracy		Precision		Stability	Repeatability
	Calibration curves	Range (μg/mL)	*R* ^2^			Recovery (%)	RSD (%)	Inter-day RSD (%)	Intra-day RSD (%)	RSD (%)	RSD (%)
GMP	Y = 784184X-187112	0.1135–7.264	0.9967	0.03	0.10	98.67	2.31	4.33	2.42	1.96	2.19
cGMP	Y = 562405X+12921	0.057–7.344	0.9969	0.02	0.05	96.94	3.79	3.86	2.01	1.75	2.95

#### 3.1.3 Detection of the relative enzyme activity of PDE5A based on a LC/MS strategy

In the process of enzyme activity determination, to ensure whether the boiling termination process affects cGMP stability, a reaction system containing only substrate was set up, and GMP was detected after boiling at 95°C for 5 min. Simultaneously, to ensure that the boiling process could completely terminate the enzyme activity, a reaction system containing only PDE5A was set up. GMP was detected after PDE5A was boiled for 5 min at 95°C and incubated with cGMP for 15 min. Finally, no GMP was detected by the LC/MS method, indicating that the boiling process not only did not affect the stability of cGMP but also completely terminated the enzymatic activity of PDE5A. To quantify the product GMP in the enzyme-catalyzed reaction, a linear standard curve of peak area and concentration was first established using the GMP standard (*R* ^2^ = 0.9967).

**FIGURE 3:**
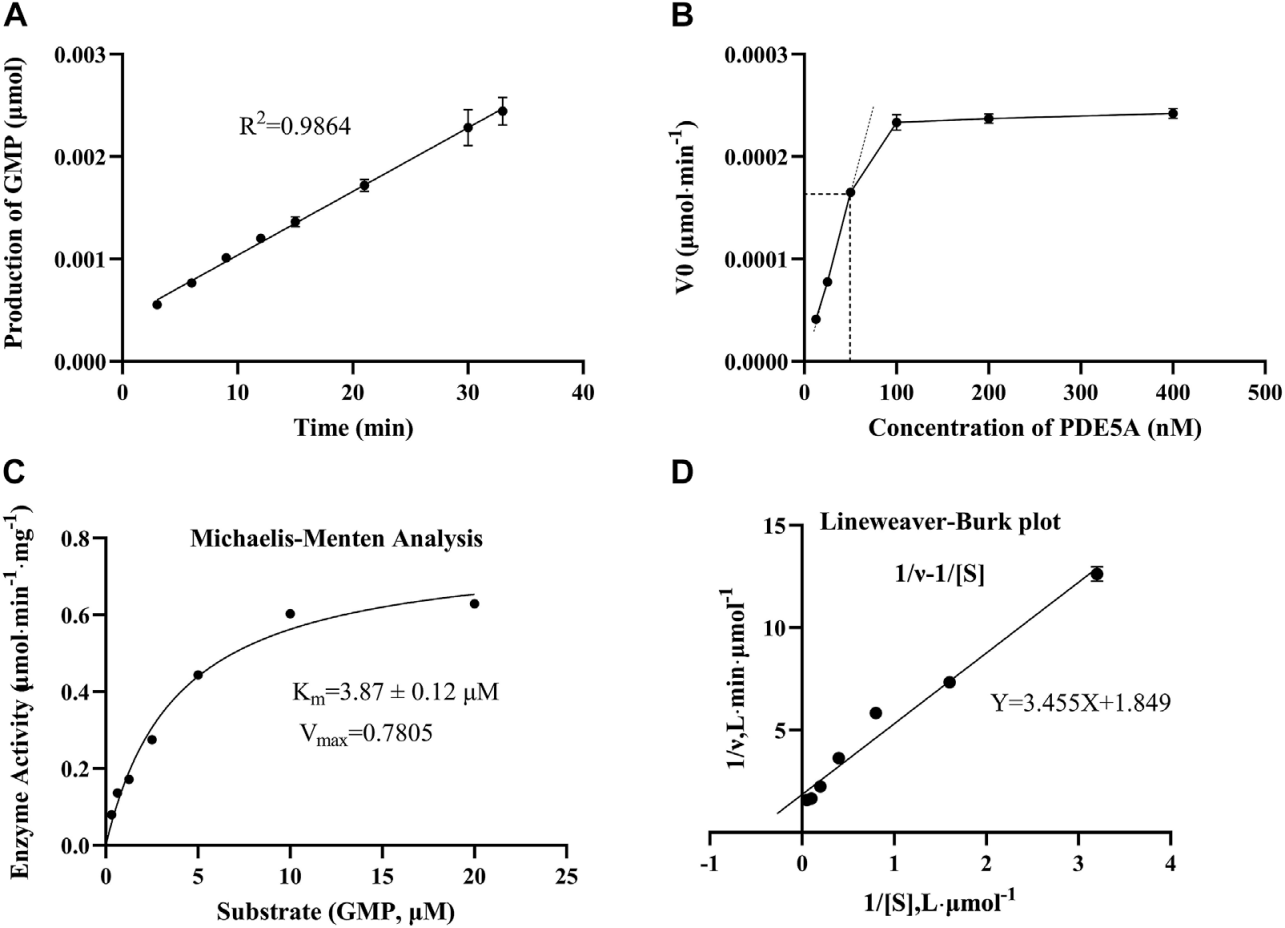
The results of the enzymatic activity assay of PDE5A. **(A)** Enzyme reaction progress curve. The linear portion of the reaction progress curve was fitted according to the throughput of GMP. **(B)** Enzyme concentration curve. The change in the initial reaction velocity (V_0_) was determined at different enzyme concentrations (C_cGMP_ = 10 μM). **(C, D)** Michaelis-Menten analysis curve and Lineweaver Burk plot. K_m_ and V_max_ were determined using progression curve analysis by varying substrate concentrations (20–0.3125 μM).

To find an optimal reaction time and quantify the enzymatic activity of PDE5A, the change in GMP was detected over time in 33 min, and a linear fit was made to the change in amount and time (Figure 3A). Finally, the optimal reaction time was selected as 15 min within the linear response range, and the absolute values of these slopes were defined as PDE5A activity. To select the appropriate enzyme concentration, we determined the initial reaction velocity (V_0_) of PDE5A at different concentrations (Figure 3B), and a concentration of 50 nM was selected within the linear range to ensure the fastest reaction rate. The specific activity was calculated by dividing the enzyme activity by the amount of enzyme, and the result was 0.3 U/mg. According to the Michaelis and Menten analysis, the half-maximum concentration (*K*_m_) of GMP was 3.87 ± 0.12 μM (Figures 3C,D), which is consistent with the reference value of the literature (Kouvelas et al., 2009). Therefore, the substrate concentration was selected to be 10 μm to ensure the enzyme binding site as much as possible (Acker and Douglas, 2014).

#### 3.1.4 Feasibility analysis of the LC/MS-based method in PDE5A inhibitor screening

**FIGURE 4:**
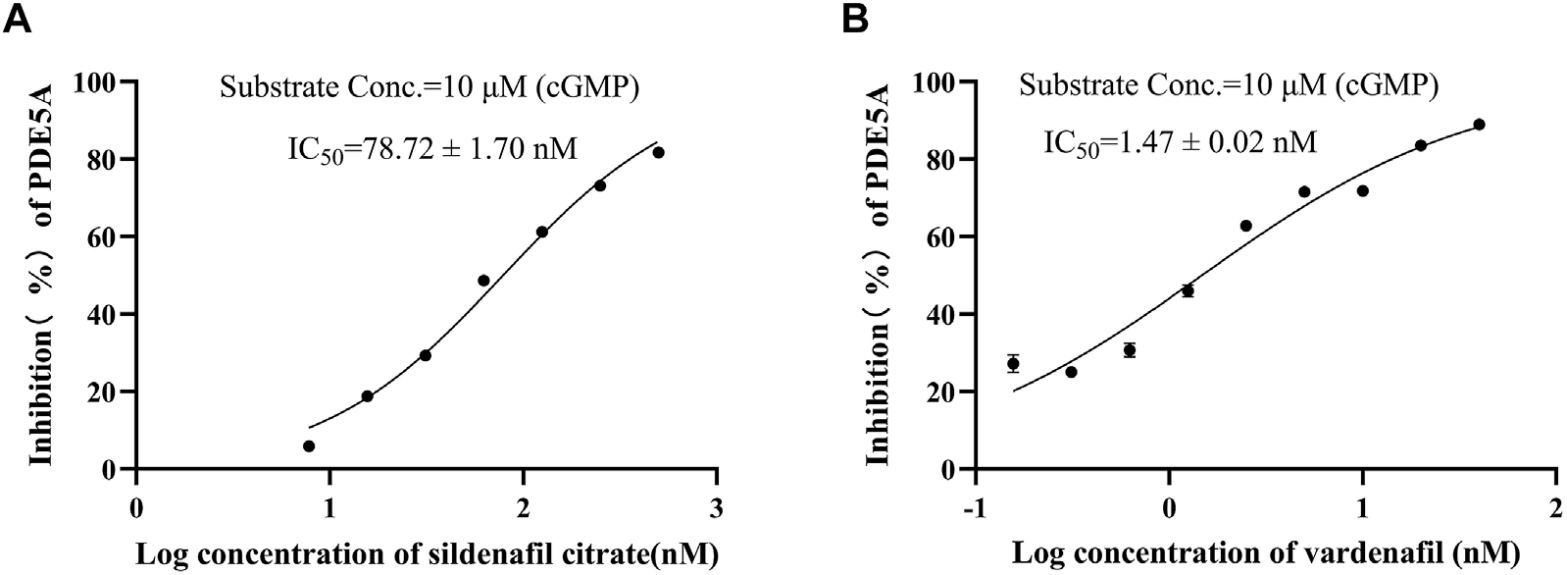
Positive drug inhibitory activity results were detected by LC/MS strategy. **(A)** Inhibitory activity of sildenafil citrate on PDE5A. **(B)** Inhibitory activity of vardenafil on PDE5A.

To show the feasibility of the developed LC/MS-based method in PDE5A inhibitor screening, this method was used to assay the inhibitory activity of sildenafil and vardenafil, which are two known inhibitors of PDE5A with high affinity for PDE5A (Shabsigh et al., 2006) and are widely used in erectile dysfunction and pulmonary hypertension. The established LC/MS-based method was used to detect the quantitative change in GMP after the addition of two inhibitors, and the inhibition rate was calculated using Equation B). Finally, sildenafil and vardenafil inhibited the enzymatic activity of PDE5A with IC_50_ values of 78.72 ± 1.7 nM and 1.47 ± 0.02 nM, respectively (Figures 4A,B), which were consistent with previous reports (Rotella 2002; Dell'Agli et al., 2005).

### 3.2 Screening of new inhibitors of PDE5A

All reported PDE5A inhibitors are competitive inhibitors, which bind to the catalytic site of PDE5A (Kim et al., 2001). Therefore, we performed a new competitive inhibitor screening based on the catalytic site of PDE5A. A total of 1,427 compounds were docked into the ligand binding site of PDE5A, and 10 compounds were finally selected for the activity assay based on the affinity score (Table 2) and visual selection. The inhibitory activities of these 10 compounds for PDE5A were determined by the LC/MS-based method and fluorescent-labeled substrates.

**TABLE 2:** Virtual screening results and essential information.

No	Name	Structure	CAS No	Docking Score (kcal/mol)
**Positive**	Sildenafil	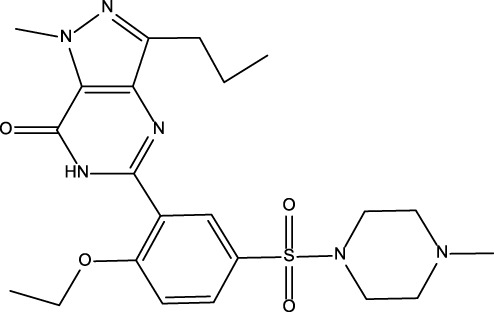	171599-83-0	−9.1
	Vardenafil	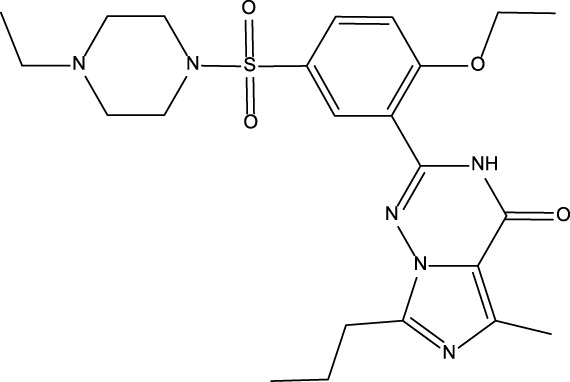	330808-88-3	−8.5
**1**	Kuwanon G	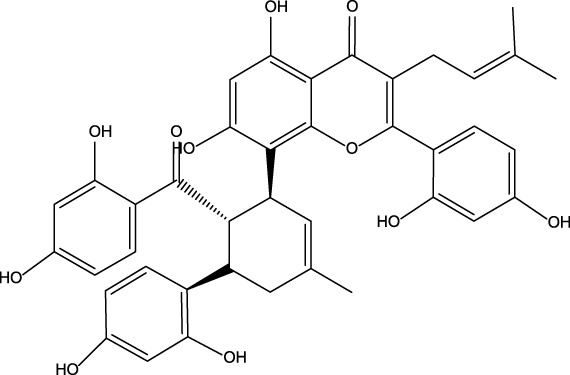	75629-19-5	−8.5
**2**	Proanthocyanidins	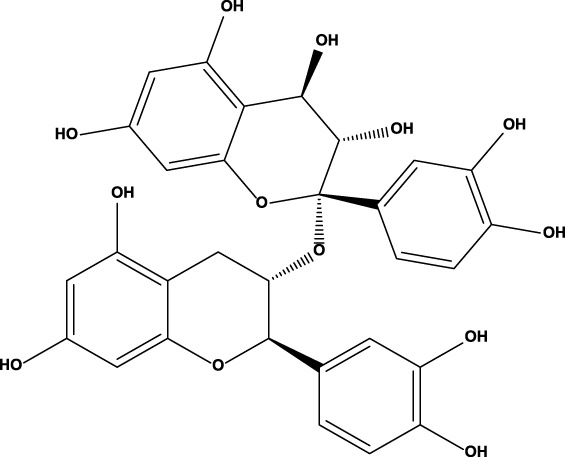	4852-22-6	−8.3
**3**	Daurisoline	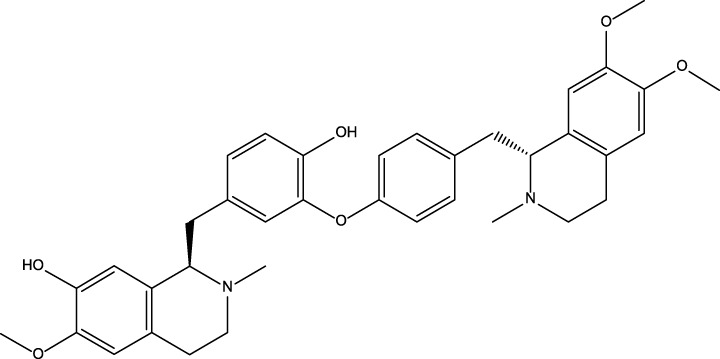	70553-76-3	−9.7
**4**	Isoliensinine	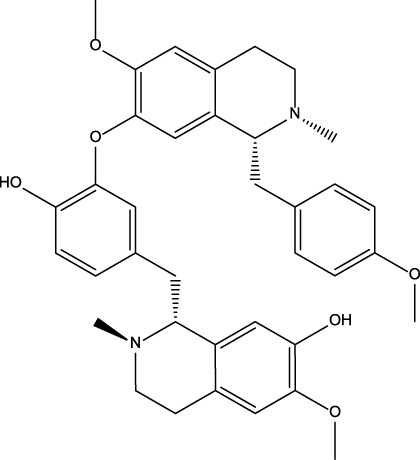	6817-41-0	−8.7
**5**	Ginkgetin	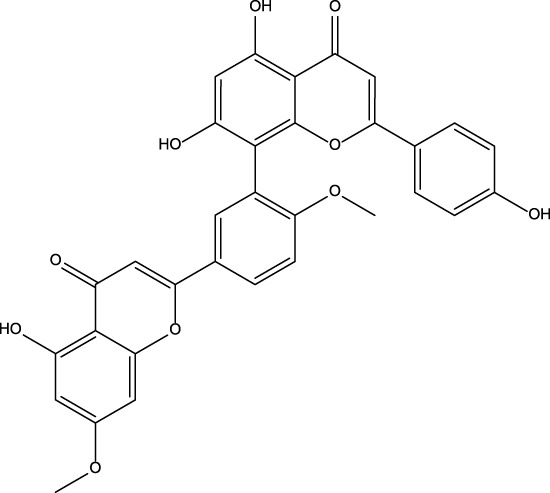	481-46-9	−10.2
**6**	7-O-Methylaloeresin A	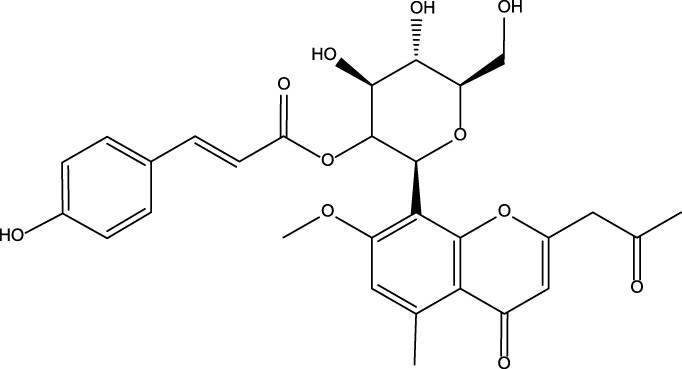	329361-25-3	−8.2
**7**	Silychristin	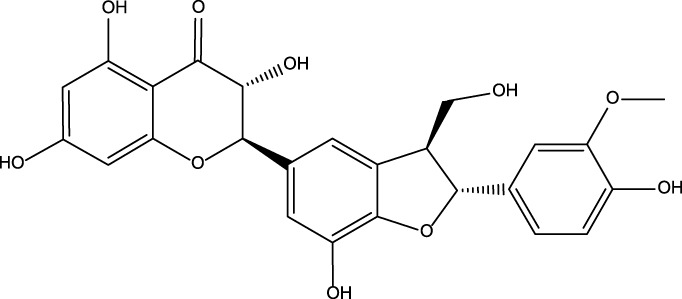	33889-69-9	−8.6
**8**	Isosilybin	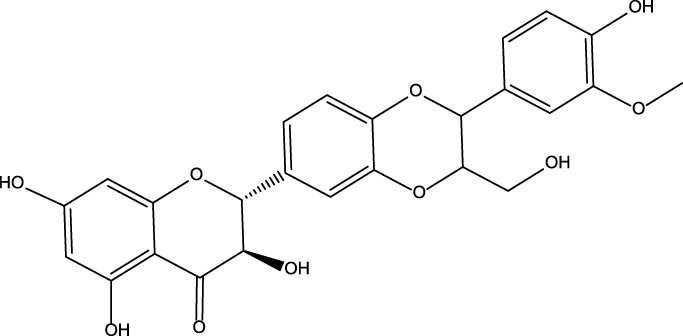	72581-71-6	−7.9
**9**	Cordycepin	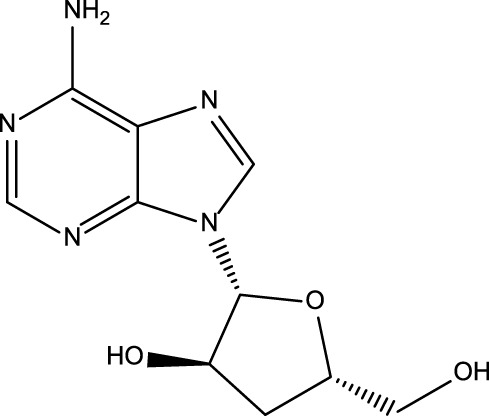	73-03-0	−7.0
**10**	Methylophiopogonone A	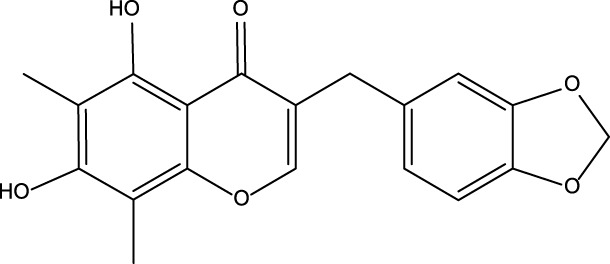	74805-90-6	−8.6

#### 3.2.1 Results of the LC/MS-based method

**FIGURE 5:**
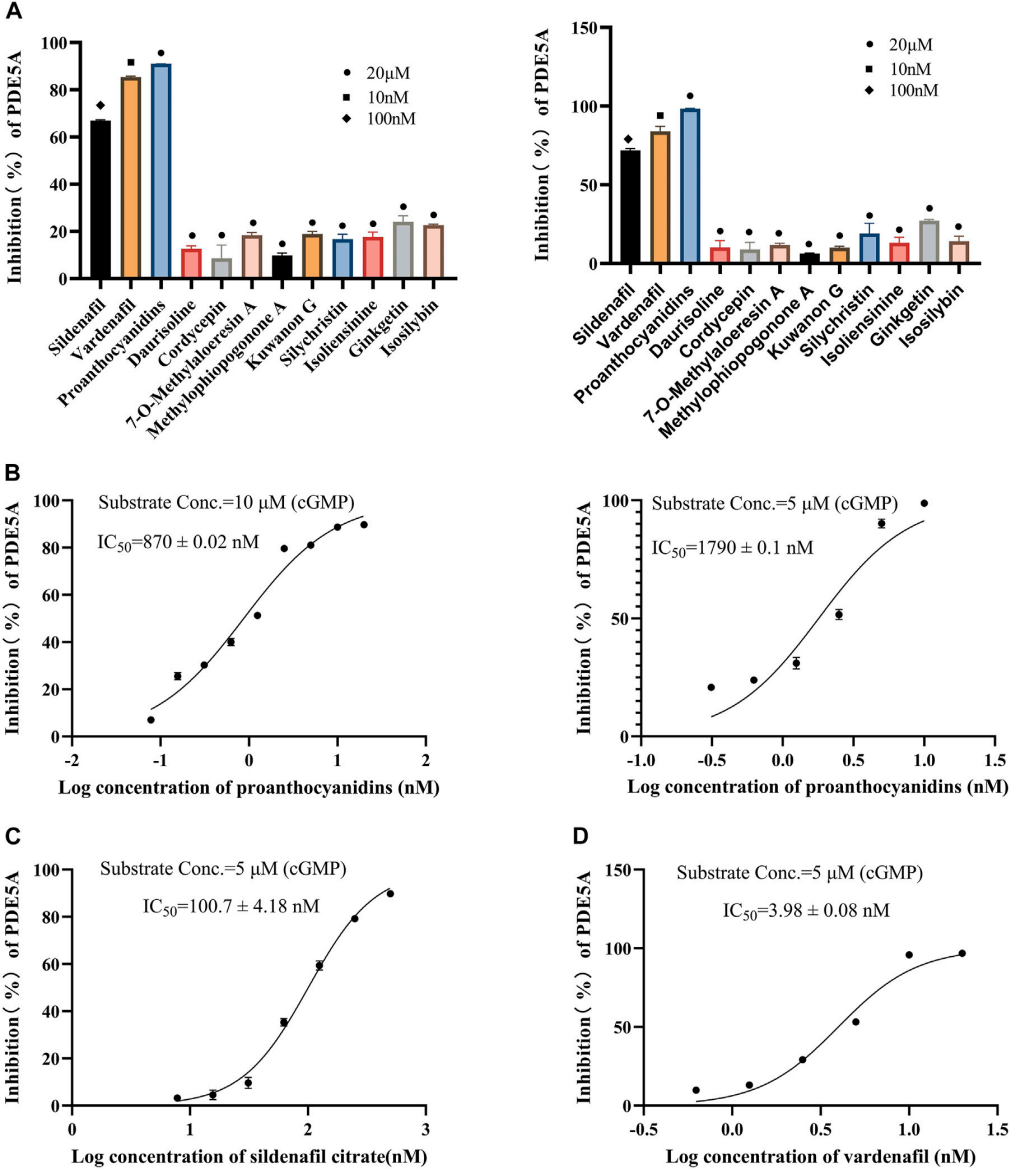
Inhibitory activity of 10 compounds for PDE5A based on the LC/MS-based method and luorescent-labeled substrate-based method. **(A)** Inhibitory activity of 10 compounds for PDE5A at 20 μΜ, and sildenafil and vardenafil were set to 100 nM and 10 nM, respectively (left: LC/MS-based method; right: luorescent-labeled substrate-based method). **(B)** Inhibition curve of proanthocyanidins. Left: LC/MS-based method; right: luorescent-labeled substrate-based method. **(C, D)** Inhibition curves of sildenafil, vardenafil (luorescent-labeled substrate-based method).

All 10 compounds from virtual screening were initially assayed at a concentration of 20 μM, and sildenafil and vardenafil were selected as positive controls. The active compounds with significant inhibition rates at this concentration were selected to further test the IC_50_. Among them, sildenafil and vardenafil exhibited 66% and 85% inhibition at 100 nM and 10 nM, respectively. Fortunately, one of the 10 compounds, named proanthocyanidins, showed significant inhibitory activity against PDE5A, with an inhibition rate of 91% at 20 μM. Other compounds were all less than 25% inhibitory (Figure 5A left). The IC_50_ of proanthocyanidins was calculated by setting gradient concentrations and was 870 ± 0.02 nΜ (Figure 5B left).

#### 3.2.2 The results of the fluorescent-labeled substrate-based method

To verify the accuracy of the LC/MS-based method, a known method based on a fluorescently labeled substrate was used to test the activity of 10 compounds. As shown in Figure 5A Right, sildenafil (100 nM) and vardenafil (10 nM) showed more than 70% inhibitory activity against PDE5A, and the inhibitory activity of proanthocyanidins (20 μM) remained the strongest among the 10 compounds and reached 98%, which was consistent with the results measured by the LC/MS-based method. The IC_50_ values of and proanthocyanidins (1790 ± 0.09 nM) were still consistent with the results measured by the LC/MS-based method (Figure 5B right), sildenafil (100.7 ± 4.18 nM), vardenafil (3.98 ± 0.08 nM, Figures 5C,D).

#### 3.2.3 Binding mode of proanthocyanidins and PDE5A

**FIGURE 6:**
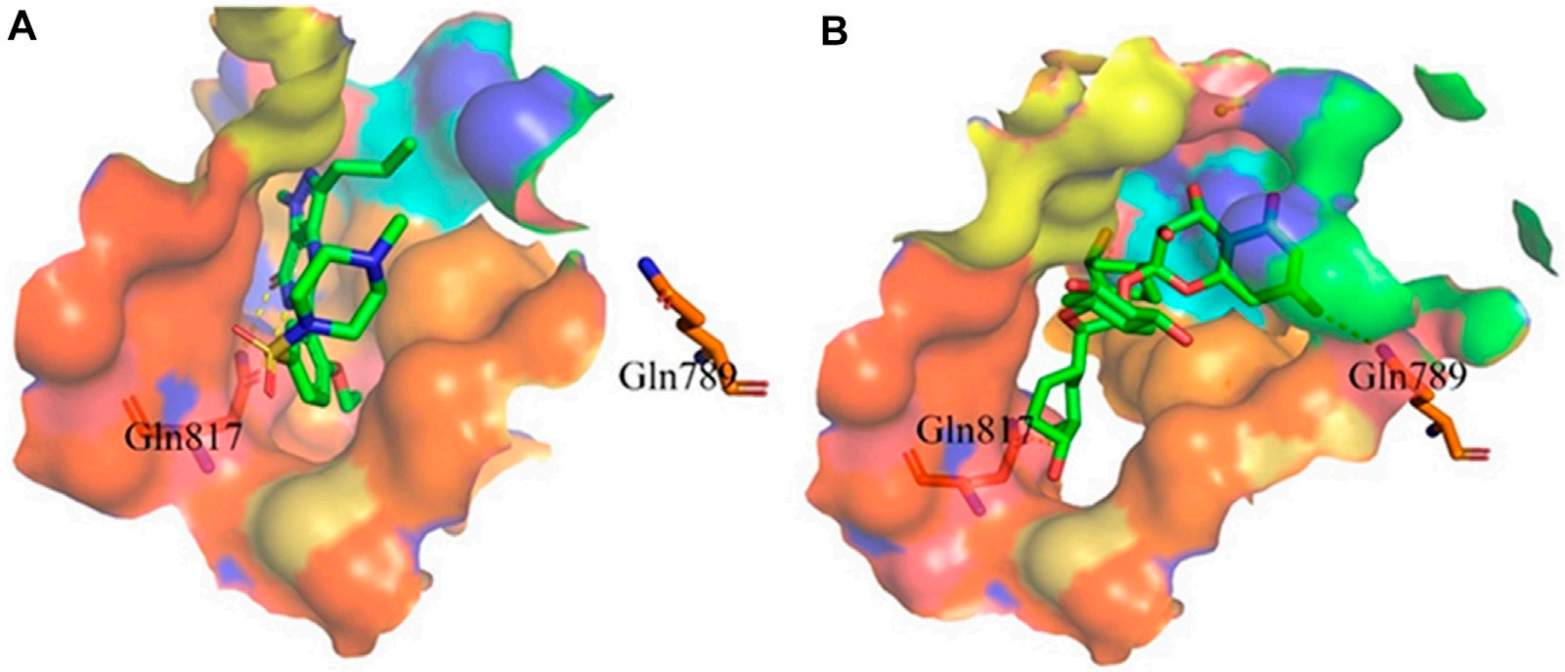
Binding mode between inhibitor and PDE5A. **(A)** Binding mode between sildenafil and PDE5A. **(B)** Binding mode between proanthocyanidins and PDE5A. Compounds are shown as a stick model with carbon atoms colored green, and PDE5A (PDB: 1TBF) is shown as the surface. Hydrogen bonds are represented by yellow dashed lines.

Proanthocyanidins, oligomeric compound formed by catechin and epicatechin molecules, are present in the flowers, nuts, fruits, bark, and seeds of various plants (Ou and Gu 2014). Here, proanthocyanidins was identified as a new inhibitor of PDE5A. To understand the inhibitory activity of proanthocyanidins, we checked the binding mode between proanthocyanidins and PDE5A based on the molecular docking result. Compared to the binding mode of sildenafil (Figure 6A), proanthocyanidins not only occupied the sildenafil site well but also a part of the structure extends to the active site on the right side and interacts with PDE5A in a hydrophobic manner (Figure 6B). Sildenafil forms a hydrogen bond with the residue Gln817, but proanthocyanidins formed another hydrogen bond with the residue Gln789 in addition to Gln817. Overall, the two-part structures of proanthocyanidins, catechin and epicatechin, not only occupy the binding site of sildenafil but also increase the binding range to the active site of PDE5, while the formation of two hydrogen bonds enhances its affinity to PDE5.

## 4 Conclusion

In conclusion, we established a new method for enzyme activity testing for PDE5A based on LC/MS, which has the advantages of being label-free, safe, and economical. Based on the high sensitivity of this method, it can detect not only the *in vitro* activity of PDE5A but also the enzyme activity of *in vivo* samples. Meanwhile, this method also provides an assay idea for enzyme activity testing based on similar substrates, such as cAMP. Moreover, proanthocyanidins was identified as a new inhibitor of PDE5A with high affinity by the LC/MS-based method.

## Data Availability

The datasets presented in this study can be found in online repositories. The names of the repository/repositories and accession number(s) can be found in the article.
